# The current incidence, prevalence, and residual risk of hepatitis B viral infections among voluntary blood donors in China

**DOI:** 10.1186/s12879-017-2861-3

**Published:** 2017-12-06

**Authors:** Ling Li, Tingting Han, Liang Zang, Libin Niu, Weifang Cheng, Hongkeng Lin, Ka Yi Li, Ruan Cao, Binghai Zhao, Yuqiang Liu, Guojin Ou, Xiao Liu, Yingjie Qi, Yanhua Li, Zhong Liu

**Affiliations:** 1 Institute of Blood Transfusion, Chinese Academy of Medical Sciences and Peking Union Medical College, Chengdu, Sichuan China; 2 No. 1 People’s Hospital of Anqing, Anqing, Anhui China; 3 Dalian Blood Center, Dalian, Liaoning China; 4 Changzhi Blood Center, Changzhi, Shanxi China; 5 Anhui Blood Center, Hefei, Anhui China; 6 Fujian Blood Center, Fuzhou, Fujian China; 7 Cooper Medical School of Rowan University, Camden, USA; 8 Mianyang Blood Center, Mianyang, Sichuan China; 9 Nanchong Blood Center, Nanchong, Sichuan China; 10 Kaifeng Blood Center, Kaifeng, Henan China; 11 No. 1 People’s Hospital of Deyang, Deyang, Sichuan China; 12 Anhui Provincial Infection Hospital, Hefei, Anhui China; 13 Department of Pathology, New York University School of Medicine, NYU Langone Health, New York, USA; 14 26 Huacai Rd, Longtan Industry Zone, Chenghua District, Chengdu, Sichuan People’s Republic of China

**Keywords:** HBV, Residual risk, Transfusion, Donors

## Abstract

**Background:**

There are few data available on the prevalence, incidence, and residual risk of transfusion-transmitted HBV (TT-HBV) infections among Chinese blood donors. This study investigated the demographic characteristics of blood donors, as well as the prevalence, incidence, and residual risk (RR) of TT-HBV infections in six large blood centers in different regions of China.

**Methods:**

The demographic characteristics and HBV screening test results of blood donors from six blood centers in different regions in China were collected and analyzed. The hepatitis B surface antigen (HBsAg) yield approach was used to estimate the incidence of HBV. Then, the RR of TT-HBV infections was evaluated using the incidence-window period model.

**Results:**

The majority of donors were between 18 and 35 years old (including 35), with the exception of the Changzhi Blood Center where a majority of donors were between 35 and 55 years old (including 55). The prevalences of HBV were 0.13%, 0.078%, 0.16%, 0.07%, 0.20%, 0.25% in Hefei, Dalian, Changzhi, Kaifeng, Mianyang and Fujian, respectively. The estimated corresponding incidences were 213.44, 161.59, 989.80, 278.05, 125.31 and 352.19 per 10^5^ person-years. Using an infectious window period of 59 days, the RR for HBV was estimated to be 34.14, 25.85, 158.35, 44.48, 20.04 and 56.35 per 10^5^ person-years in Hefei, Dalian, Changzhi, Kaifeng, Mianyang and Fujian, respectively.

**Conclusion:**

Despite the introduction of more sensitive assays in blood screening, our data revealed that the current residual risk of TT-HBV infection was still high (overall 56.53 per 10^5^ py). A continuous monitoring of the residual risk of transfusion-transmitted infections is crucial for safe blood management.

## Background

Blood donor screening practices for the hepatitis B virus (HBV) infections vary from country to country both in terms of the manufacturers of the reagents and the testing methods. Over the past 30 years, the risk of TT-HBV has markedly decreased due to the development of more sensitive hepatitis B surface antigen (HBsAg) tests, the introduction of screening for antibodies against the hepatitis B core antigen (anti-HBc) in some countries, the use of nucleic acid tests (NAT), and improved volunteer donor recruitment processes [1–3]. In recent years, the government of China has taken several measures to improve the safety of the blood supply, which included promulgating a new blood donation law in 1998 [4] and revising standard protocols for donor screening and donation screening processes in 2012 [5]. However, in China, the risk of TT-HBV still remains higher than that of other routinely screened viruses, such as HCV and HIV. When using only HBsAg tests for HBV infection screening, the risk of TT-HBV is high because donors may appear HBsAg-negative but are actually HBV infected, such as those currently in the window period (WP) or at the late stage of infection. This is especially true for the areas with both a high prevalence of HBV and a lack of NAT screening [6]. Also, there is an additional risk associated with chronic OBI. OBI is usually defined (in the blood donor screening context) as an HBV infection without detectable HBsAg, usually presenting itself as anti-HBc positive and, typically, with low levels of HBV DNA. Liang et al. reported that the prevalence of HBsAg in the general population in China fell to 7.2% from 9.8% after the implementation of vaccination against hepatitis B [7], which has played an important role in decreasing the rate of HBV infection.

The residual risk (RR) is different with different blood screening strategies. Using NAT for donor screening can shorten the window period and identify occult HBV infections (OBI) which cannot be detected by HBsAg tests [6]. In terms of the risk for TT-HBV, it is a problem particularly in the countries and/or areas with both a high prevalence of HBV and where NAT for HBV is not used routinely for donor screening [8]. Despite the implementation of donor screening by NAT, there are still a number of countries where there is a residual risk for TT-HBV [9–11].

China still is a developing country with most areas undeveloped economically. At present, most blood centers cannot afford NAT, thus most donations are routinely screened with two ELISA tests for HBsAg in China. This issue has been brought to the attention of the relevant authorities of the Chinese Government, which have decided to pilot NAT testing for HBV, HCV, and HIV in all provincial blood centers in 2015 during blood donor screening [12]. This study aims to evaluate the residual risk (RR) of HBV infections in China before the implementation of NAT. This study will provide helpful data to assess the effectiveness of the implementation of NAT in China. Available data about the prevalence, incidence, and RR of HBV infections among Chinese blood donors is limited. In 2013, Wuping Li and his colleagues reported their findings on the prevalence, incidence, and residual risk of HBV infections in the Anhui Blood Center from 2009 to 2011 [13]. However, that study was based on the data from one single blood center, which is the limitation of that study.

**Fig. 1:**
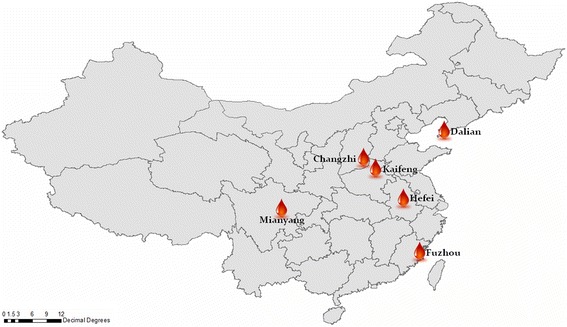
Geographic Distribution of the Six Blood Centers and the Institute of Blood Transfusion

The present study involves six blood centers located in different regions of China (Fig. 1).Hopefully this study was therefore more representative. The aims of the present study were to evaluate the current prevalence, incidences and RR of HBV infections of blood donors among the six blood centers, and to provide guidance for developing and monitoring evidence-based blood donor management strategies to improve the safety of the blood supplies regarding the RR of TT-HBV infections.

## Methods

### Ethics statement

This study was approved by the Ethics Committee of the Institute of Blood Transfusion, of the Chinese Academy of Medical Sciences & Peking Union Medical College. Written informed consent was obtained from each study participant before the interview, sample collection and testing.

### Participants and study procedure

This study was a collaborative effort between the Institute of Blood Transfusion (IBT) of the Chinese Academy of Medical Sciences and six blood centers located in different regions of China. The six Chinese regional blood centers were the Anhui Blood Center (Hefei, Anhui, located in the east), Fujian Blood Center (Fuzhou, Fujian, located in the south), Dalian Blood Center (Dalian, Liaoning, located in the north), Changzhi Blood Center (Changzhi, Shanxi, located centrally), Kaifeng Blood Center (Kaifeng, Henan, located centrally) and Mianyang Blood Center (Mianyang, Sichuan, located in the west). Figure 1 shows the geographic distribution of the six blood centers. The study population consisted of all blood donors who donated at one of the six participating blood centers or at one of their mobile blood collection vehicles between July 1, 2014 and June 30, 2015. All blood donations were screened with the serological tests for HIV, HBV, HCV and syphilis. All samples of HBsAg reactive screening were sent to the IBT national reference laboratory for blood donor testing, and then were subjected to the HBsAg neutralization test and anti-HBc tests. Residual risks could be calculated by the HBV yield approach as Li et al. described [13].

### History questionnaire and rapid pre-donation screening

Following the “Technical and Operational Guidelines and Procedures for Blood Centers” issued by the Chinese Ministry of Health on December 31, 2011 [12], all six blood centers had the same approach for blood donor screening, requiring all blood donors to pass a routine pre-donation screening process that consisted of a medical history questionnaire, a brief physical examination, and pre-donation rapid screening. The medical history questionnaire included questions about their histories of sexually transmitted diseases, hepatitis, illegal parenteral drug use, sex with multiple partners, and men who had sex with men (MSM). If any of the above items screened positive, the donors were permanently deferred. The physical examination included body temperature, body weight, and blood pressure. Before blood collection, all donors underwent rapid testing at the collection sites for the hepatitis B surface antigen (HBsAg, Rapid Test Kit, Aikang Bio-technology Co., Ltd., Hangzhou, China) as well as rapid testing for Alanine Aminotransferase (ALT) (ALT Rapid Test Kit, Rongsheng Biological Pharmaceutical Co., Ltd., Shanghai, China) and hemoglobin (Hb) (Hemoglobin Assay Kit, Amyjet Scientific Inc., Wuhan, China). The donors with increased ALT levels or a reactive HBsAg result would be temporarily deferred, the samples and information were saved if donors tested HBsAg positive on the rapid test.

### Routine screening tests for the HBV infection

All successful donations were subjected to tests on two different HBsAg assays by an enzyme-linked immuno sorbent assay (ELISA). If both testing on the two different HBsAg assays were reactive, the screening test was defined as reactive. If the results from either one of two tests was reactive, the sample was retested in duplicate on the same assay and/or another appropriate kit. The screening test was defined as reactive if either one or two positive reactive results were obtained during retesting of the HBsAg ELISA tests. If both tests showed non-reactive results, the screening test was defined as non-reactive, and the corresponding donation was qualified for transfusion.

All test kits were approved and licensed by the Chinese State Food and Drug Administration (or Food and Drug Administration, FDA). The reagents used for donor screening tests are listed in Table 1, and the assays were performed following the manufacturer’s instructions.

**Table 1:** Screening Test Kits for HBsAg Used at Each Blood Center

Blood Center	First Assay		Second Assay	
	Test Kit	Sensitivity (%) / Specificity (%) / Limit (ng/ml)	Test Kit	Sensitivity (%) /Specificity (%) / Limit (ng/ml)
Anhui	WANTAI (Beijing)	99.5 / 98.5 / 0.03	BIO-RAD (United States)	100 / 97.55 / ND
Fujian	BIO-RAD (United States)	100 / 97.55 / ND	WANTAI (Beijing)	99.5 / 98.5 / 0.03
Dalian	InTec (Xiamen, China)	100 / 98.6 / ND	BIO-RAD (United States)	100 / 97.55 / ND
Changzhi	BIO-RAD (United States)	100 / 97.55 / ND	InTec (Xiamen, China)	100 / 98.6 / ND
Kaifeng	BIO-RAD (United States)	100 / 97.55 / ND	WANTAI (Beijing)	99.5 / 98.5 / 0.03
Mianyang	WANTAI (Beijing)	99.5 / 98.5 / 0.03	BIO-RAD (United States)	100 / 97.55 / ND

*ND* no data

*WANTAI* Beijing WANTAI Biological Pharmacy Enterprise Co., Ltd.

*BIO-RAD* BIO-RAD Clinical Diagnostics

*InTec* Xiamen InTec Biological Pharmacy Enterprise Co., Ltd.

### Confirmation testing

**Fig. 2:**
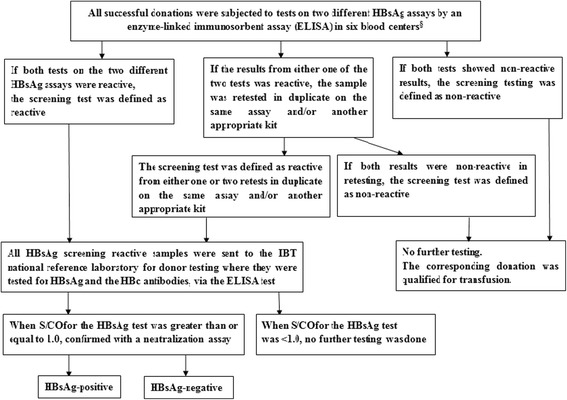
Testing Algorithm. Note: §: We do not take into account the risk from OBI. HBsAg: Hepatitis B surface Antigen. HBc antibody: Hepatitis B core antibody. ELISA: Enzyme-Linked Immuno Sorbent Assay. IBT: Institute of Blood Transfusion. S/CO: the signal to cutoff ratio

All HBsAg screening reactive samples, including pre-donation rapid screening and after-donation reactive testing by ELISA, were sent to the IBT national reference laboratory for donor testing where they were tested for HBsAg, via the ELISA test (MONOLISA TM HBsAg ULTRA, BIO-RAD, California, USA), and anti-HBc antibodies (HBcAb ELISA Kit, Beijing WANTAI Biological Pharmacy Enterprise Co. Ltd., Beijing, China). When the signal to cutoff ratio (S/CO) for the HBsAg test was greater than or equal to 1.0, the ELISA for HBsAg tests were considered as reactive, after which they were confirmed with a neutralization assay (Reagent Kit for the Confirmation of the HBV Surface Antigen, ZHUHAI LIVZON DIAGNOSTICS INC., Zhuhai, China). If there was a positive confirmed result by neutralisation, the sample was confirmed as HBsAg-positive; if a negative neutralisation result was obtained, the samples were considered HBsAg-negative. The reagents used for donor screening tests in IBT are listed in Table 2, and the testing algorithm is listed in Fig. 2 *.*

**Table 2:** Screening Test Kits Used for IBT

Kit Name	Company	Sensitivity / Specificity (%)
Diagnostic Kit for HBV	BIO-RAD (United States)	100 / 97.55
Surface Antigen (ELISA)		
HBsAg Neutralization,	Livzon (Zhuhai)	no data
Antibody to Hepatitis B		
Surface Antigen (Human)		
Diagnostic Kit for	WANTAI (Beijing)	99.4 / 99.1
Antibody to Hepatitis		
Core Antigen (ELISA)

The demographic characteristics of all donors among the six blood centers were collected and analyzed (Table 3) according to and including donor status (first time donors vs repeat donors), gender, age, occupation and education. First-time donors were defined as donors who had no record on file according to the blood center databases. Repeat donors were donors who had a previous record in the databases of the blood centers.

**Table 3:** Demographic Characteristics of all Donors Across Six Blood Centers from June, 2014 to July, 2015

Blood Centers	Anhui	Dalian	Changzhi	Kaifeng	Mianyang	Fujian
Donors Status						
First-Time	60,812(62.26%)	38,503(49.75%)	10,643(32.43%)	40,227(67.11%)	18,889(41.52%)	43,384(51.92%)
Repeat	36,856(37.74%)	38,892(50.25%)	22,174(67.57%)	19,711(32.89%)	26,605(58.48%)	40,170(48.08%)
Gender						
Male	65,105(66.66%)	48,496(62.66%)	20,790(63.35%)	38,942(64.97%)	27,742(60.98%)	50,124(59.99%)
Female	32,563(33.34%)	28,899(37.34%)	12,027(36.65%)	20,996(35.03%)	17,752(39.02%)	33,430(40.01%)
Age Group						
18~25	42,212(43.22%)	32,597(42.12%)	2701(8.23%)	11,688(19.50%)	9313(20.47%)	28,450(34.05%)
25~35	27,328(27.98%)	21,421(27.68%)	7269(22.15%)	15,740(26.26%)	10,150(22.31%)	21,933(26.25%)
35~45	18,762(19.21%)	15,439(19.95%)	13,199(40.22%)	19,234(32.09%)	16,005(35.18%)	20,989(25.12%)
45~55	8907(9.12%)	7536(9.74%)	8765(26.71%)	11,976(19.98%)	9276(20.39%)	10,854(12.99%)
55~60	459(0.47%)	402(0.52%)	883(2.69%)	1301(2.17%)	751(1.65%)	1329(1.58%)
Occupation						
Farmers	5801(5.94%)	3243(4.19%)	12,270(37.39%)	16,405(27.37%)	5687(12.50%)	2565(3.07%)
Workers	10,206(10.45%)	12,027(15.54%)	5907(18.00%)	11,286(18.83%)	4841(10.64%)	2423(2.90%)
Students	23,020(23.57%)	18,103(23.39%)	1414(4.31%)	5580(9.31%)	4599(10.11%)	14,647(17.53%)
Soldiers	1845(1.89%)	859(1.11%)	98(0.30%)	623(1.04%)	641(1.41%)	1504(1.80%)
Teachers	2724(2.79%)	410(0.53%)	755(2.30%)	468(0.78%)	1210(2.66%)	/
Civil Servants	3437(3.52%)	186(0.24%)	1195(3.64%)	336(0.56%)	1843(4.05%)	4278(5.12%)
Doctors	4189(4.29%)	573(0.74%)	597(1.82%)	2242(3.74%)	3071(6.75%)	3175(3.81%)
Staff	24,026(24.60%)	2748(3.55%)	2110(6.44%)	22,914(38.23%)	3662(8.05%)	19,518(23.36%)
Other	22,414(22.95%)	39,247(50.71%)	8470(25.81%)	84(0.14%)	19,940(43.83%)	35,444(42.42%)
Education						
Below High School	18,645(19.09%)	22,174(28.65%)	15,765(48.04%)	21,799(36.37%)	14,185(31.18%)	31,458(37.65%)
High School and	42,662(43.68%)	21,733(28.08%)	14,407(43.90%)	30,784(51.36%)	22,893(50.32%)	23,111(27.66%)
Associate Degree						
Bachelor’s Degree	22,044(22.57%)	20,726(26.78%)	2543(7.75%)	5556(9.27%)	7670(16.86%)	28,525(34.14%)
Master’s Degree	3340(3.42%)	805(1.04%)	30(0.09%)	1091(1.82%)	669(1.47%)	460(0.55%)
Others	10,977(11.24%)	11,958(15.45%)	72(0.22%)	707(1.19%)	77(0.17%)	/
Sum up	97,668	77,395	32,817	59,938	45,494	83,554
Total	396,866					

HBsAg-positive donors and/or donations were defined by neutralization assay. The prevalence of HBV infections was calculated by the number of HBsAg positive donations divided by the number of total donations.

The incidence rate of HBV was determined by using the HBsAg yield approach as previously described [10]. In the process of the HBV infection, HBsAg becomes detectable earlier than anti-HBc. HBsAg may be transient, while anti-HBc can persist for a long time. Therefore, donations confirmed as HBsAg-positive but non-reactive for anti-HBc can be regarded as newly infected cases that are considered to be HBsAg yield cases. The number of HBsAg yield cases divided by the total number of donations is equal to the HBsAg yield rate. The quotient divided by the length of time a patient has been HBsAg-positive prior to anti-HBc seroconversion (termed the HBsAg yield window, calculated as 44 days) gives an incidence estimate for HBV infections among blood donors [14].

The RR attributable to WP donations was calculated using the following equation:

$$ \mathrm{HBsAg}\ \mathrm{new}\ \mathrm{infection}\  \mathrm{rate}=\mathrm{Number}\  \mathrm{of}\  \mathrm{HBsAg}\  \mathrm{positive}\ \mathrm{but}\ \mathrm{anti}-\mathrm{HBc}-\mathrm{negative}/\mathrm{The}\  \mathrm{total}\  \mathrm{number}\  \mathrm{of}\  \mathrm{donations}; $$

the ratio of HBsAg new infection rate = HBsAg new infection rate of first − time donors over HBsAg new infection rate of repeat donors;

$$ \mathrm{Incidence}\  \mathrm{rate}\  \mathrm{for}\ \mathrm{HBV}\ \mathrm{infection}=\mathrm{HBsAg}\ \mathrm{new}\ \mathrm{infection}\  \mathrm{rate}/\mathrm{HBsAg}\  \mathrm{yield}\  \mathrm{window}\ \left(44\ \mathrm{days}\right); $$

$$ \mathrm{RR}=\mathrm{incidence}\  \mathrm{rate}\  \mathrm{for}\ \mathrm{HBV}\ \mathrm{infection}\times \mathrm{window}\  \mathrm{period}\ \left(0.16\ \mathrm{years}\  \mathrm{or}\ 59\ \mathrm{days}\right); $$

Data were statistically analyzed using computer software (SPSS 17.0, SPSS, Chicago, IL). An approximate 95% confidence interval (95% CI) was obtained using the Poisson distribution model. The Chi-square test was performed to assess the association between the categorical variants. A *P*-value of <0.05 was used as the cut-off level for significance.

## Results

### Demographic characteristics of blood donors

From July 1, 2014 to June 30, 2015, a total of 558,089 blood donations were collected at six blood centers located in different areas of China *(see* Figure 1). The demographic characteristics of all of the donors at the six blood center were collected, and the distributions of all demographic characteristics were examined *(see* Table 3). Among the Anhui, Dalian and Fujian Blood Centers, almost 60% of all donations came from donors aged between 18 and 35 years old, while almost 40% at Changzhi, Kaifeng and Mianyang blood centers were of this age group. Across all of the blood centers, the proportions of male and female donors were 63% and 37%, respectively. Donors with a high school education or less contributed to 70% of all donations. Overall, more than half of the donors were employees and students. There were significant differences in the constitutions of donors in terms of their demographic characteristics across the six blood centers in this study.

### Serologic prevalence of HBV infections confirmed by the neutralization assay

In this study there were 558,089 donor samples, of which there were 1664 donor samples found to be HBsAg reactive. All of the 1664 samples were tested for anti-HBc and HBsAg and confirmed by neutralisation, and those that were HBsAg positive and anti-HBc positive totaled 484, while those that were HBsAg positive and anti-HBc negative totaled 94; those that were HBsAg negative and anti-HBc positive totaled 326, and those that were HBsAg negative and anti-HBc negative totaled 760. 746 of the 1664 were repeat donors, the mean of interdonation interval for repeat donors was about 11.9 months, and the interdonation interval data for all 746 repeat donors is shown in Table 4 *.*

**Table 4:** The interdonation interval data for all 746 repeat donors

The interdonation interval (month)	6-9(including 9)	9-12	12-15	15-18	18-21	21-24	>24 Total
Donors	339	194	62	34	41	57	19,746
Mean(month)	11.9						

Overall, the serologic prevalence of confirmed HBV infections from first-time and repeat donations is shown in Tables 5 and 6. In all, 578 samples were HBsAg positive, confirmed by neutralization assay. The overall prevalence was 0.15%. The seroprevalence of HBsAg was estimated to be 0.13%, 0.078%, 0.16%, 0.07%, 0.20%, and 0.25% at the Hefei, Dalian, Changzhi, Kaifeng, Mianyang and Fujian Blood Centers, respectively. The prevalence of HBsAg varied significantly among the six blood centers, and the prevalence in Fujian was much higher (0.25%) than all of the other blood centers. By contrast, the prevalence in Kaifeng was the lowest (0.07%) among all of the blood centers. Of all 578 reactive samples, 520 (98.5%, 520/528) were from first-time donors.

**Table 5:** HBsAg Prevalence by Blood Center / Bank Among all Donors (First-Time and Repeat)

Blood Center	Donations (Total)	First-Time			Repeat			Prevalence(%) (Among all Donors)
		Donations	Number of HBsAg Confirmed Positive	Prevalence (%) (95% Confidence Intervals)	Donations	Number of HBsAg Confirmed Positive	Prevalence (%) (95% Confidence Intervals)	
Hefei	97,668	60,812	115	0.19 (0.15-2.23)	36,856	10	0.027 (0.01-0.04)	0.13 (0.11-0.15)
Dalian	77,395	38,503	52	0.14 (0.09-0.17)	38,892	8	0.02 (0.01-0.04)	0.08 (0.06-0.09)
Changzhi	32,817	10,643	40	0.38 (0.26-0.49)	22,174	12	0.05 (0.02-0.08)	0.16 (0.12-0.20)
Kaifeng	59,938	40,227	36	0.09 (0.06-0.12)	19,711	4	0.02 (0.004-0.04)	0.07 (0.05-0.09)
Mianyang	45,494	18,889	77	0.41 (0.32-0.50)	26,605	12	0.05 (0.02-0.07)	0.20 (0.16-0.24)
Fujian	83,554	43,384	200	0.46 (0.40-0.52)	40,170	12	0.03 (0.013-0.05)	0.25 (0.22-0.29)
Total	396,866	212,458	520	0.24 (0.22-0.27)	184,408	58	0.03 (0.02-0.04)	0.15 (0.13-0.16)

**Table 6:** HBV Prevalence for Blood Donors Above and Below 35 Years Old

Blood Center	Donations	Below 35			Over 35			*P*-value	Total Prevalence
		Donations	Number of HBsAg confirmed positive	Prevalence	Donations	Number of HBsAg Confirmed Positive	Prevalence		
Hefei	97,668	69,540	38	0.06%	28,128	87	0.31%	<0.001	0.13%
Dalian	77,395	54,018	16	0.03%	23,377	44	0.19%	<0.001	0.08%
Changzhi	32,817	12,671	11	0.09%	20,146	41	0.20%	0.01	0.17%
Kaifeng	59,938	27,428	12	0.04%	32,510	28	0.09%	0.045	0.07%
Mianyang	45,494	19,463	21	0.11%	26,031	68	0.26%	<0.001	0.20%
Fujian	83,554	50,383	55	0.11%	33,171	157	0.47%	<0.001	0.25%
Total	396,866	233,503	153	0.07%	163,363	625	0.38%	<0.001	0.20%

### Estimated incidence of HBV infection using the HBsAg yield approach

In order to estimate HBV incidence accurately, an HBsAg yield window of 44 days was used as described in a previous study [11]. The ratio of HBsAg new infection rate of donations from first-time donors to repeat donors was 6.97, 6.57, 6.94, 4.41, 9.04 and 15.43, at the Hefei, Dalian, Changzhi, Kaifeng, Mianyang and Fujian Blood Centers, respectively. Overall, the estimated incidence rate was 213.44, 161.59, 989.80, 278.05, 125.31 and 352.19 (per 100,000 person-years) in Hefei, Dalian, Changzhi, Changzhi, Kaifeng, Mianyang and Fujian, respectively *(see* Table 7).

**Table 7:** HBsAg Incidence by Blood Center/ Bank Among First-Time and Repeat Donors

Blood Center	First-Time		Repeat			Ratio of^a^ HBsAg new infection rate	Yield Window (year)	Incidence (per 10^5^ py)
	Number of HBsAg Confirmed Positive, Anti-HBc Negative	Incidence (per 10^5^ py)	Number of HBsAg Confirmed Positive, Anti-HBc Negative	Yield Rate (per 10^5^ py)	Incidence (per 10^5^ py)			
Hefei	13	315.39	2	5.43	45.25	6.97	0.12	213.3
Dalian	36	281.52	2	5.14	42.85	6.57	0.12	161.59
Changzhi	5	2347.34	9	40.59	338.23	6.94	0.12	989.80
Kaifeng	10	372.89	2	10.15	84.56	4.41	0.12	278.05
Mianyang	2	261.14	5	3.47	28.89	9.04	0.12	125.31
Fujian	6	640.19	2	4.98	41.19	15.43	0.12	352.19
Total	72	773.49	22	11.93	99.42	7.78	0.12	197.38

^a^The HBsAg new infection rate in repeat donors is the HBsAg+/HBc antibody- prevalence, and the incidence for HBV infections in first-time donors is equal to the incidence for HBV infections in repeat donors multiplied by the ratio of HBsAg new infection rate (the ratio of HBsAg new infection rate = HBsAg new infection rate of first-time donors over HBsAg new infection rate of repeat donors). The overall incidence for HBV infections in all donors is the percentage of incidence in first-time donors plus the percentage of incidence in repeat donors. For Hefei, 315.18 × 10^−5^ × 0.6226 (Number of first-times / number of first-times + number of repeats = 60,812 / 97,668) + 45.22 × 10^−5^ × 0.3774 = 196.23 × 10^−5^ + 17.07 × 10^−5^ = 213.3 × 10^−5^)

The residual risk calculation model used in this study is one of the more suitable computational models in the absence of nucleic acid detection. Based on the results of the last two blood tests, it is possible to extrapolate whether the recent donors have a new infection. However, for the first-time donors, the new infection ratio cannot be determined or calculated directly. Therefore, this model is adopted to indirectly establish the incidence and residual risk of the first-time donors

### RR estimates obtained using the HBsAg yield approach

The residual risk of HBV infection was estimated by applying the refined infectious window-period estimate of 0.16 years (or 59 days) [15] to the derived incidence estimates. The residual risk of HBV infection among blood donations from the participating blood centers is shown in Table 8 *.* The estimated residual risks were 34.14, 25.85, 158.35, 44.48, 20.04 and 56.35 (per 100,000 person-years) in Hefei, Dalian, Changzhi, Kaifeng, Mianyang and Fujian, respectively. The residual risk of Dalian was the highest, approximately 6 times that of Fujian, which had the lowest residual risk.

**Table 8:** HBsAg Residual Risk by Blood Center/ Banks Among First-Time and Repeat Donors

Blood Center	First-time		Repeat		Window Periods in Year	Overall (per 10^5^ py)
	Incidence (per 10^5^ py)	Residual risk (per 10^5^ py)	Incidence (per 10^5^ py)	Residual risk (per 10^5^ py)		
Hefei	315.39	50.46 (6.13-182.04)	45.25	7.24 (0.88-26.12)	0.16	34.14
Dalian	281.52	45.04 (5.46-162.62)	42.85	6.85 (0.83-24.75)	0.16	25.85
Changzhi	2347.34	375.57 (64.40-721.85)	338.23	54.12 (9.28-104.01)	0.16	158.35
Kaifeng	372.89	59.66 (7.22-215.38)	84.56	13.53 (1.64-48.84)	0.16	44.48
Mianyang	261.14	41.78 (3.98-65.31)	28.89	4.62 (0.44-7.22)	0.16	20.04
Fujian	640.19	102.43 (12.39-369.78)	41.19	6.59 (0.80-23.96)	0.16	56.35
Average	703.08	112.49 (15.04-187.96)	96.83	15.49 (1.93-24.16)	0.16	56.53

## Discussion

In China, most donations were routinely subjected to screening for HBsAg by ELISA twice. The remaining few blood centers screen HBV infection by ELISA(×1) plus NAT. However, due to the transient nature of the HBsAg detectable period during the HBV infection process, and also due to the lack of routine screening testing for anti-HBc antibodies, the residual risk of HBV infection has remained high in China [8, 13, 16]. In order to develop an evidence-based, efficient and safe blood donor screening strategy and/or policies to decrease the RR of HBV infections, it is essential to have epidemiological information regarding the prevalence, incidence and RR of HBV infections and the associated demographic characteristics of both high-risk and low-risk populations of voluntary blood donors. The demographic characteristics of volunteer blood donors in different blood centers in China were quite different. For example, the percent of the first time donors among the six blood centers ranged from 32.43% to 67.11% of all donations. The proportion of young donors 18 to 25 years old was 43.22% of all donations at the Anhui Blood Center, but only 8.23% at the Changzhi Blood Center. Meanwhile, the proportion of middle aged donors 35 to 55 years old was 28.33% of all donations at the Anhui Blood Center, but was 66.93% at the Changzhi Blood Center. There were also some differences in the occupations and education levels at the different blood centers. For instance, 37.39% of donors were farmers, 18.00% were workers and 4.31% were students at the Changzhi Blood Center, while these proportions were 3.07%, 2.9% and 17.53%, respectively, at the Fujian Blood Center. 91.94% of donors had either received an Associate’s degree, a high school diploma or below at the Changzhi Blood Center, while this figure was 56.68% at the Dalian Blood Center. However, some similarities were noted among different blood centers regarding the gender of the donors; namely male donors comprised the majority of donors (with a mean of 61%) at all of the blood centers. In addition, more than half of all donors at all of the blood centers were under 35 years old in this study except for those at the Changzhi Blood Center (see Table 3).

The differences found in the demographic characteristics at different blood centers may have contributed to the differences of estimated residual risks at the blood centers. A higher RR was found at Changzhi (158.35 per 100,000 donations per year), while the lowest was found at Mianyang (20.04 per 100,000 donations per year) (see Table 7). According to the characteristics of the blood donors, most of the blood donors were first-time donors, and only Changzhi had the most repeat blood donors, reaching 67.57%. Theoretically, and according to published data, repeat donors have a lower RR for infectious diseases [12, 14]. However, the results of this study showed that the HBV incidence and residual risks at Changzhi were the highest. With regards to the age of the blood donors, most of them were under the age of 35, but most (70%) donors at Changzhi were over 35 years old. China began nationwide hepatitis B vaccinations in 1992, so the residual risk of donors under 35 years old is lower than that of donors above 35 years old. Perhaps this is thus a major cause of Changzhi having the highest residual risk. This data also proved that the HBV vaccine in China has been successful, and it shows that hepatitis B vaccination can effectively reduce residual risks. Second, the proportion of farmers at the Changzhi Blood Center was 37.39%, much higher than the proportions at the other five blood centers. (These proportions ranged from 3.07% at the Fujian Blood Center to 27.37% at the Kaifeng Blood Center. See Table 3). Due to economic barriers and other multivariate factors, farmers had the lowest vaccination rate, which again could have led to their higher susceptibility to HBV infections. Third, the proportion of donors without a high school education was relatively high at the Changzhi Blood Center. These donors may have had a relatively low level of health knowledge, which also could have accounted for their elevated rates of HBV infection. The findings in this study are very interesting, because the evidence above indicates that the evaluation of residual risks should take into consideration not only the proportions of first time and repeat donors, but also their ages, occupations and education backgrounds.

TT-HBV can still occur after transfusion, even though the carrier blood has tested negative for HBsAg. Apart from occult HBV infections, there remains a limitation in the model that was used to estimate the residual risk of HBV, because neither the window period nor the incidence of donor HBV infections is precisely known. Thus, theoretically, the actual infection risk may be underestimated, which might not accurately reflect infectivity. However, the estimated result of Li’s model can still be used to evaluate the safety of blood supplies.

Some countries and/or regions have low prevalence of HBV infections, such as Canada and Hong Kong. These two locations exhibit low residual risks of 1:1,700,000 [17] and 1:22,000 [9], respectively, whereas China has a moderate-to-high residual risk for HBV infections (about 1:13,670 in Shanghai) [18]. Although the prevalence of HBsAg in the Chinese population has dropped to 7.2% in 2006 from 9.8% since the implementation of the nationwide HBV vaccination program in 1992, the prevalence of HBV infections in China is still high [7, 8, 13, 18]. Continued efforts are still needed in donor education, improving donor recruitment and screening strategies. The incidence window period model used in this study can also be used to evaluate the potential impact of HBV NAT implementation by calculating the expected percentage residual risk reduction and yield of a particular assay system [19]. Per the request of the Chinese Ministry of Health, NAT testing for HBV, HCV, and HIV will have been piloted in all provincial blood centers by 2015 [9]. This may decrease the residual risk of TT-HBV markedly.

## Limitations

This study may underestimate the incidence of HBV infections due to its definition of an infection incident and because its risk estimates do not take into account the risk from OBI. The numbers of HBsAg positive and anti-HBc negative tests in each region are low, leading to a general lack of precision in RR calculations (see the wide 95% CIs). In terms of limitations, the incidence calculations are sensitive to assumptions around the window period. In addition, the study may have a number of other limitations around window period estimates. Although, this study included six different locations of blood centers from a diversity of geographical areas, it still may not be representative of all of China.

## Conclusion

Despite the introduction of more sensitive assays in blood donor screening, our data revealed that the current residual risk of transfusion-transmitted HBV infection is still high (overall 56.53 per 10^5^ py). To improve the safety of blood supplies, we need to continue to educate blood donors and improve donor recruitment and screening strategies. NAT for blood donor screening is needed in China, which may markedly decrease the RR for HBV infections and improve the safety of blood supplies. It is worth considering testing for anti-HBc during blood donor screening in China.

## Abbreviations

ALT: Alanine aminotransferase
ELISA: Enzyme-linked immuno sorbent assay
HBc antibody: Hepatitis B core antibody
HBsAg: Hepatitis B surface antigen
NAT: Nucleic acid tests
OBI: Occult HBV infection
RR: Residual risk
S/CO: The signal to cutoff ratio
TTHBV: Transfusion-Transmitted HBV
WP: Window period
